# Behavioral Responses Associated with a Human-Mediated Predator Shelter

**DOI:** 10.1371/journal.pone.0094630

**Published:** 2014-04-09

**Authors:** Graeme Shannon, Line S. Cordes, Amanda R. Hardy, Lisa M. Angeloni, Kevin R. Crooks

**Affiliations:** 1 Department of Fish, Wildlife, and Conservation Biology, Colorado State University, Fort Collins, Colorado, United States of America; 2 Graduate Degree Program in Ecology, Colorado State University, Fort Collins, Colorado, United States of America; 3 Department of Biology, Colorado State University, Fort Collins, Colorado, United States of America; University of California, Berkeley, United States of America

## Abstract

Human activities in protected areas can affect wildlife populations in a similar manner to predation risk, causing increases in movement and vigilance, shifts in habitat use and changes in group size. Nevertheless, recent evidence indicates that in certain situations ungulate species may actually utilize areas associated with higher levels of human presence as a potential refuge from disturbance-sensitive predators. We now use four-years of behavioral activity budget data collected from pronghorn (*Antilocapra americana*) and elk (*Cervus elephus*) in Grand Teton National Park, USA to test whether predictable patterns of human presence can provide a shelter from predatory risk. Daily behavioral scans were conducted along two parallel sections of road that differed in traffic volume - with the main Teton Park Road experiencing vehicle use that was approximately thirty-fold greater than the River Road. At the busier Teton Park Road, both species of ungulate engaged in higher levels of feeding (27% increase in the proportion of pronghorn feeding and 21% increase for elk), lower levels of alert behavior (18% decrease for pronghorn and 9% decrease for elk) and formed smaller groups. These responses are commonly associated with reduced predatory threat. Pronghorn also exhibited a 30% increase in the proportion of individuals moving at the River Road as would be expected under greater exposure to predation risk. Our findings concur with the ‘predator shelter hypothesis’, suggesting that ungulates in GTNP use human presence as a potential refuge from predation risk, adjusting their behavior accordingly. Human activity has the potential to alter predator-prey interactions and drive trophic-mediated effects that could ultimately impact ecosystem function and biodiversity.

## Introduction

Predators impact prey through two key processes, firstly by directly killing and removing individuals from the population and secondly through the indirect effects of predation risk that result in prey species modifying their behavior [1], [2]. These non-lethal effects have a strong influence on prey fitness with evidence suggesting substantial impacts at the population level possibly equal to or greater than the removal of individuals through direct predation [3]–[5]. Furthermore, the risk of predation varies across time and space with herbivores constantly balancing foraging effort against the need for safety from predators [6]–[9]. Prey species therefore inhabit ranges of shifting predation risk that has been termed the ‘ecology or landscape of fear’ [2], [10], [11]. The landscape of fear is specific to the prey species and will depend on the predators to which they are exposed, the encounter rate, predatory defense and the effectiveness of vigilance [1], [2], [12].

Recently, there has been considerable interest in the role of predation risk in regulating ecosystems through trophic cascades [3], [5], [13], including most notably the reintroduction of wolves (*Canis lupus*) to Yellowstone National Park in 1995 [10], [11], [14]. Interestingly, human disturbance in natural and protected areas has been shown to affect wildlife populations in a similar manner to predation risk, including greater rates of movement and vigilance, reduced foraging, shifting habitat use and increases in group size [15]–[19]. These behavioral responses result in potential fitness costs as a result of increased energy expenditure, loss of foraging opportunities and the direct impact of physiological stress on reproductive success and survival [15], [20]. However unlike predation risk, anthropogenic disturbance in protected areas exhibits greater spatial and temporal predictability, being largely a function of visitors using defined park infrastructure (e.g. roads and trail networks) during daylight hours.

Large-bodied carnivores such as wolves and bears often demonstrate the greatest sensitivity to human presence [17], [21], [22], whereas a number of large herbivore species have exhibited much greater tolerance of human activities and infrastructure [19], [23]–[25]. A primary reason why herbivores are predicted to exhibit greater tolerance to human presence in natural areas is that it provides a potential shelter from risk-sensitive carnivores. For example, female moose (*Alces alces*) in the Yellowstone Ecosystem showed a distinct preference for areas close to roads during their highly synchronous calving period because these zones functioned as refugia from human-averse brown bears (*Ursus arctos*) [26]. Meanwhile, two recent North American studies demonstrated that large herbivores were more likely to use areas in close proximity to roads and walking trails compared with carnivore species, and by doing so were experiencing a spatial refuge from potential predators [27], [28]. Disturbance driven by human activities might therefore alter community-level interactions between predators and prey [22], [29].

Although research has begun to indicate that ungulate species may select areas that have elevated levels of human use, much less attention has been focused on the specific behavioral outcomes associated with potential predator shelters. We now use a four-year behavioral dataset collected from the direct observation of elk (*Cervus elephus*) and pronghorn (*Antilocapra americana*) to explore the potential refuge effect generated by tourist infrastructure in Grand Teton National Park (GTNP). Based on the ‘predator shelter hypothesis’, we predicted that the Teton Park Road in GTNP, which is associated with significantly higher levels of traffic and human activity than our comparison site, the quieter River Road, would provide ungulates with the opportunity to trade off human disturbance against a shelter from predation. More specifically, we predicted that enhanced predation risk at the River Road would result in pronghorn and elk forming larger groups due to the benefits afforded by safety in numbers [30]–[32]. The specific behavioral responses mediated by the predator shelter were predicted to include an increase in the proportion of individuals feeding and a reduction in the proportion of animals engaged in alert behavior [2], [6], [7], concomitant with the lower perceived levels of predatory risk.

## Materials and Methods

### Study site

The study was conducted in Grand Teton National Park (GTNP), northwestern Wyoming, USA, from June to October over a four-year period (2007–2010). The park covers an area of approximately 1250 km^2^ and is characterized by mountainous topography with rugged steep slopes and flat valley bottoms. Our research focused on two parallel sections of road running north to south along the eastern base of the Teton Range: the Teton Park Road, a 22 km section of paved two lane road, and the 27 km unpaved River Road, accessible only by four-wheel-drive vehicles and running along the Snake River approximately 2–5 km east of the Teton Park Road. The Teton Park Road is a main route via which tourists explore the park and as such it receives high levels of use compared with the quieter River Road. The traffic volume and overall levels of human activity is the key difference between the two study sites (30-fold greater at the Teton Park Road), with vegetation and habitat structure being highly similar and dominated by open sage grassland (95% of the behavioral scans were conducted in this vegetation type). The valley through which both roads run is key summer and autumn habitat for migrating herbivores including elk, pronghorn, moose and mule deer (*Odocoileus hemionus*), whilst the resident predator species include grizzly bears, black bears (*Ursus americanus*), wolves, coyotes (*Canis latrans*), mountain lions (*Puma concolor*) and bobcats (*Lynx rufus*). Grand Teton National Park, including S. Cain and S. Dewey, granted permission to conduct this research.

### Behavioral observation

Behavioral data were collected from elk and pronghorn, the two ungulate species that were most prevalent along the sections of road in our study area. To collect behavioral data, two observers conducted 1–2 road surveys per day between sunrise and sunset during the field season from June-October. This resulted in a total of 376 surveys over the four-year study (mean number of surveys per year 94±12 SE). Each survey took from 2–4 hours, while the behavioral scan data for an individual group was collected over a period of 5–15 minutes. During the Teton Park Road surveys, the field vehicle stopped at 42 pre-assigned scan points that were approximately 160–800 m apart and offered the maximum view of the surrounding landscapes while ensuring that groups of animals were not double counted. Rough substrate on the River Road required slower speeds, allowing the observer to scan open terrain for wildlife and stop at fewer scan points where visible terrain was maximized. These approaches enabled the observers to accurately document each group of ungulates along the two sections of road. The total number of tourist vehicles (moving and stationary) within 200 m of the scan point was recorded before behavioral observations of ungulate groups were conducted.

A group of ungulates was defined as ≥1 individual present, and a distance of 100 m was used to delineate different herds, following [33] who described this as the maximum distance at which elk respond to conspecific vocalizations. The time, location, species and the number of individuals were recorded for each group. A single observer then scanned the group from left to right noting the instantaneous behavior of each individual using a spotting scope. The behaviors used for the analyses were classified as feeding (stationary grazing), alert (scanning with head at or above shoulder level, and/or displaying vigilance toward a particular stimulus) and moving (walking or running with no evidence of foraging), with the remaining behaviors (bedding, mating and defensive) categorized as other. The distance of the group to the road was recorded as a categorical variable using discrete 100 m distance bins. The maximum distance at which ungulate groups could be detected and observed (∼1 km) was the same for both sites due to the predominantly open and relatively flat terrain of the Teton valley.

### Data analysis

We conducted preliminary analyses on the differences between the Teton Park Road and the River Road, firstly by determining if there was a significant difference in the number of tourist vehicles recorded during behavioral scans at each site. The second stage of the analysis compared the average group size between sites for each species in order to test the prediction that average group sizes of elk and pronghorn were smaller at the Teton Park Road due to the predator shelter effect generated by elevated human activity. Generalized linear models were used for these analyses with site as the single explanatory variable. We used a quasi-poisson model to account for overdispersion in the elk group size analysis. The research vehicle was not included in these initial analyses as we intended to compare typical traffic volumes between the roads; the research vehicle was commonly the only vehicle present at many of the scan points along the River Road.

The behavioral scan data for pronghorn and elk were then analyzed using generalized linear mixed modeling. A set of models was constructed to predict the probability that each individual exhibited a given behavior, expressed as a bivariate response variable (i.e., 1 or 0 corresponding to whether or not each individual was feeding, alert and moving). The models incorporated the explanatory variables relating to a human-mediated predator shelter: *site* (Teton Park Road vs River Road), *distance to road*, *number of vehicles*, and *group size*. The distance to road variable was categorized on the basis of animal groups being <200 m, 200–500 m and >500 m from the road, following approaches used in previous research on herbivores [18], [33], [34]. The <200 m distance to road category was used as the level by which the other two distance classes were compared in all analyses. The number of vehicles was a count of passing and stationary traffic recorded during the behavioral scan. The research vehicle was included in this variable to account for any effect of the observers on ungulate behavior.

We selected candidate models *a priori* for each behavioral response variable that incorporated several potential explanatory variables: *site* (Teton Park Road vs. River Road) to test for behavioral differences between locations predicted by the predator shelter hypothesis; *group size* and *number of vehicles*, which were expected to differ between the two sites with potential effects on perceived risk; and *distance to road* and its interaction with *site* to determine whether any predator shelter effects of the Teton Park Road might wane with distance. Thus, our set of eight candidate models included the null model, a site only model, two additive models (site + group size; site + number of vehicles), a model incorporating the additive effect of site and distance to road as well as the interaction between the two (site + distance to road + site * distance to road), two models built on the latter including the additive effect of group size or number of vehicles (site + distance to road + group size + site * distance to road; site + distance to road + number of vehicles + site * distance to road), and finally the global model (site + distance to road + group size + number of vehicles + site * distance to road). A binomial error distribution was used for all models since all response variables were included as a series of successes and failures, depending upon whether an individual engaged in a specific behavior. Residual plots were used to confirm the fit of the models.

All individual observations from scanning a single ungulate group were given a herd ID that was distinct from the herd IDs of other scans. This herd ID was incorporated as a random effect in the model to account for the possible correlation between behaviors within a herd (following the approach of Brown et al. [24]). However, it is important to note that animals within the study were not individually recognizable and the same individuals could potentially have been sampled on multiple occasions. Model selection was performed using Akaike' Information Criterion adjusted for small sample size (AICc) [35] in conjunction with model weights since several models were very close in AICc score. Analyses were carried out within the lme4 package in R (v. 2.15.1; R Core Development Team 2012). Due to the close proximity of AICc scores between models, we present the top models accounting for ≥0.95 of the AICc weight. The effect size (β-estimates) of individual parameters was extracted from these models. The significance of the parameter was assessed by whether the 95% confidence intervals of the β-estimates overlapped zero, in which case there was no effect of the parameter in question.

## Results

**Figure 1:**
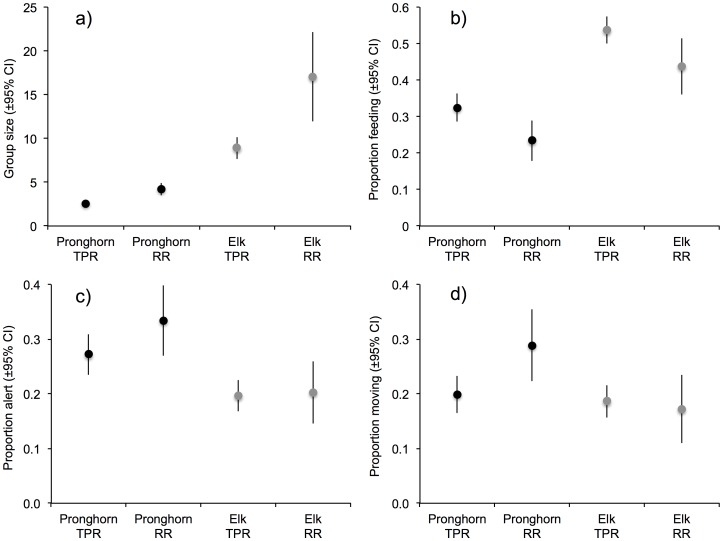
Comparisons of the average group size (a), and proportion of pronghorn and elk engaged in feeding (b), alert behavior (c) and movement (d) at the Teton Park Road and the River Road. Data are presented as means ±95% CI.

Traffic levels differed significantly between the two sites (β-estimate  = 1.21, 95% CI  = 1.07/1.35) with an average of 2.6 (±0.11 SE) vehicles per observation at the Teton Park Road and 0.1 (±0.02 SE) vehicles per observation at the River Road. During the four-year study, 1040 behavioral scans and 6553 animal observations (Teton Park Road: 3707 elk, 1061 pronghorn; River Road: 1226 elk, 559 pronghorn) were conducted on 493 groups of elk and 547 groups of pronghorn. On average per road survey, we observed 19.7 (±SE 1.9) elk in 2.2 (±0.1 SE) groups and 4.3 (±0.2 SE) pronghorn in 1.7 (±0.1 SE) groups along the Teton Park Road, and we observed 37.2 (±6.3 SE) elk in 2.2 (±0.2 SE) groups and 10.3 (±1.1 SE) pronghorn in 2.4 (±0.2 SE) groups along the River Road. The average group size was smaller at the Teton Park Road than the River Road for both pronghorn (β-estimate  = −0.49, 95% CI  = −0.59/−0.39) and elk (β-estimate  = −0.65, 95% CI  = −0.93/−0.35; Figure 1a).

The results of the behavioral scan analysis are presented in Table 1 for pronghorn and Table 2 for elk. We identified three top models predicting pronghorn feeding behavior, accounting for 0.95 of the AICc weight (Table 1). The β-estimates and confidence intervals extracted for each parameter in the best models revealed that pronghorn, as predicted, were more likely to feed at the Teton Park Road compared to the River Road (27% increase in proportion feeding, see Figure 1b). Pronghorn also fed more when in larger groups (Table 1). Moreover, the interaction between site and distance to road demonstrated that pronghorn were more likely to be observed feeding close to the Teton Park Road (<200 m) compared to distances further away (200–500 m and >500 m distance categories). At the River Road the opposite effect was observed, with greater levels of feeding further from the road (Table 1). The main effect of distance to road suggests an apparent overall increase in feeding >500 m from a road, however this appears to be an artifact of the interaction as demonstrated by the lack of an effect in a simple additive model that did not include an interaction term (β-estimate_200-500m_  = −0.03, 95% CI  = −0.41/0.34; β-estimate_>500m_  = −0.03, 95% CI  = −0.57/0.52).

**Table 1:** Top models accounting for 0.95 of the AICc weight in the pronghorn behavioral analyses.

	K	ΔAICc	AICc weight	Site (TPR)	Dist. to road (200–500 m)	Dist. to road (>500 m)	Group size	No. Vehicles	Site*Dist. (200–500 m)	Site*Dist. to (>500 m)
**Feeding**										
Site + Dist. + Group size + Site*Dist. Road	8	0.00	0.54	**1.41 (0.67/2.15)**	0.69 (−0.11/1.49)	**1.30 (0.29/2.30)**	**0.08 (0.04/0.13)**		**−0.93 (−1.83/−0.02)**	**−1.89 (−3.09/−0.69)**
Site + Group size	4	1.52	0.25	**0.63 (0.22/1.04)**			**0.08 (0.04/0.13)**			
Site + Dist. road + Group size + Vehicles + Site*Dist. road	9	1.96	0.20	**1.38 (0.61/2.14)**	0.69 (**−**0.11/1.49)	**1.30 (0.29/2.30)**	**0.08 (0.04/0.13)**	0.01 (**−**0.06/0.09)	**−0.93 (−1.83/−0.02)**	**−1.89 (−3.09/−0.69)**
**Alert**										
Site	3	0.00	0.24	**−0.41 (−0.78/−0.05)**						
Site + Dist. road + Site*Dist. Road	7	0.51	0.19	**−0.97 (−1.57/−0.38)**	**−**0.65 (**−**1.31/0.01)	**−**0.85 (**−**1.75/0.04)			0.69 (**−**0.09/1.48)	**1.42 (0.33/2.51)**
Site + Group size	4	0.81	0.16	**−0.47 (0.84/−0.09)**			**−**0.02 (**−**0.06/0.02)			
Site + Dist. road + Group size + Site*Dist. road	8	1.32	0.13	**−1.05 (−1.65/−0.44)**	**−0.67 (−1.32/−0.01)**	**−**0.86 (**−**1.75/0.04)	**−**0.02 (**−**0.07/0.02)		0.73 (**−**0.05/1.51)	**1.41 (0.33/2.49)**
Site + Vehicles	4	2.02	0.09	**−0.42 (−0.83/−0.01)**				0.01 (**−**0.07/0.07)		
Null	2	2.52	0.07							
										
Site + Dist. road + Vehicles + Site*Dist. Road	8	2.54	0.07	**−0.99 (−1.61/−0.37)**	**−**0.65 (**−**1.31/0.01)	**−**0.85 (**−**1.75/0.05)		0.01 (**−**0.07/0.08)	0.69 (**−**0.09/1.48)	**1.42 (0.33/2.51)**
**Moving**										
Site	3	0.00	0.45	**−0.73 (−1.28/−0.18)**						
Site + Vehicles	4	0.84	0.30	**−0.88 (−1.50/−0.26)**				0.06 (**−**0.05/0.18)		
Site + Group size	4	2.03	0.16	**−0.73 (−1.29/−0.17)**			0.00 (**−**0.07/0.07)			
Null	2	4.53	0.05							

β-estimates (±95% CI) are given for each model parameter with bold text denoting 95% CI that do not overlap zero. K is the parameter count for the model (including intercept and variance).

**Table 2:** Top models accounting for 0.95 of the AICc weight in the elk behavioral analyses.

	K	ΔAICc	AICc weight	Site (TPR)	Dist. to road (200–500 m)	Dist. to road (>500 m)	Group size	No. Vehicles	Site*Dist. (200–500 m)	Site*Dist. to (>500 m)
**Feeding**										
Site + Group size	4	0.00	0.47	**0.67 (0.24/1.10)**			**0.01 (0.00/0.02)**			
Site	3	0.65	0.34	**0.61 (0.19/1.04)**						
Site + Vehicles	4	2.58	0.13	**0.64 (0.19/1.09)**				−0.01 (−0.05/0.03)		
Null	2	6.26	0.02							
										
**Alert**										
Site + Dist. road + Group size + Site*Dist. road	8	0.00	0.31	−**0.84 (**−**1.66/**−**0.02)**	−**1.12 (**−**2.04/**−**0.19)**	−**1.55 (**−**2.51/**−**0.59)**	−0.01 (−0.01/0.00)		0.89 (−0.12/1.89)	**1.31 (0.27/2.35)**
Site + Dist. road + Site*Dist. road	7	0.32	0.26	−**0.84 (**−**1.67/0.00)**	−**1.14 (**−**2.08/**−**0.20)**	−**1.66 (**−**2.63/**−**0.69)**			0.90 (−0.13/1.93)	**1.42 (0.36/2.47)**
Site + Dist. road + Vehicles + Site*Dist. road	8	1.66	0.13	−0.76 (−1.61/0.08)	−**1.13 (**−**2.07/**−**0.19)**	−**1.66 (**−**2.62/**−**0.69)**		−0.02 (−0.05/0.02)	0.89 (−0.13/1.91)	**1.39 (0.33/2.44)**
Site + Dist. road + Group size + Vehicles + Site*Dist. road	9	1.91	0.12	−0.78 (−1.61/0.06)	−**1.10 (**−**2.02/**−**0.18)**	−**1.54 (**−**2.50/**−**0.58)**	−0.01 (−0.01/0.00)	−0.01 (−0.05/0.03)	0.86 (−0.14/1.87)	**1.29 (0.25/2.33)**
Null	2	2.86	0.07							
										
Site + Group size	4	3.10	0.06	0.06			−0.01			
				(−0.32/0.45)			(−0.02/0.00)			
**Moving**										
Null	2	0.00	0.37							
										
Site + Vehicles	4	1.08	0.22	0.04 (−0.62/0.70)				0.05 (−0.01/0.11)		
Site	3	1.60	0.17	0.21 (−0.41/0.82)						
Site + Dist. road + Vehicles + Site*Dist. road	8	3.26	0.07	−0.54 (−1.88/0.81)	−0.41 (−1.90/1.08)	−**1.61 (**−**3.18/**−**0.04)**		0.05 (−0.01/0.10)	0.13 (−1.49/1.75)	1.35 (−0.35/3.05)
Site + Dist. road + Site*Dist. road	7	3.47	0.07	−0.35 (−1.67/0.96)	−0.40 (−1.89/1.08)	−**1.60 (**−**3.17/**−**0.04)**			0.12 (−1.49/1.74)	1.28 (−0.41/2.97)
Site + Group size	4	3.64	0.06	0.20 (−0.42/0.83)			0.00 (−0.01/0.01)			

β-estimates (±95% CI) are given for each model parameter with bold text denoting 95% CI that do not overlap zero. K is the parameter count for the model (including intercept and variance).

For pronghorn alert behavior, seven models accounted for ≥0.95 of the AICc weight and were within 3 AICc scores (Table 1). As predicted, pronghorn were less likely to be alert at the Teton Park Road compared to the River Road (18% reduction in proportion alert, see Figure 1c). The interaction between site and distance to road was driven by alert behavior increasing markedly >500 m from the Teton Park Road. In the pronghorn movement behavior analysis, four models within five AICc scores accounted for ≥0.95 of the AICc weight (Table 1). Only site had β-estimates with confidence intervals that did not overlap zero, indicating that pronghorn were more likely to be moving at the River Road compared with the Teton Park Road (30% increase in proportion moving, see Figure 1d).

Model selection for the analysis of elk feeding behavior generated four top models within seven AICc scores (Table 2). Consistent with predictions, elk were more likely to feed at the Teton Park Road in comparison to the River Road (21% increase in proportion feeding, see Figure 1b). For alert behavior, six models accounted for 0.95 of the AICc weight and were within four AICc scores (Table 2). As predicted, elk were less alert at the Teton Park Road compared to the River Road (Table 2), however the proportionate data demonstrate that this 9% reduction was comparatively weak (Figure 1c). Elk were more alert >500 m from the Teton Park Road compared to the closest distance category, while the converse relationship was evident at the river road (Table 2). Similar to the pronghorn feeding analysis, the inclusion of the interaction term is likely driving the main effect of alert behavior decreasing with distance, as demonstrated by the reduced strength of the effect in a simple additive model (β-estimate_200-500m_  = −034, 95% CI  = −0.71/0.03; β-estimate_>500m_  = −0.43, 95% CI  = −0.80/−0.06). The results for movement behavior of elk included six top models that accounted for 0.95 of the AICc weight and were within four AICc scores (Table 2). This analysis did not reveal any site-specific differences in movement (Table 2, Figure 1d), with the null model receiving the most support from the data.

## Discussion

Our results suggest that human activity can alter ungulate behavior in accordance with the predator shelter hypothesis. More specifically, as predicted under the predator shelter hypothesis, elk and pronghorn formed smaller groups, were more likely to forage and less likely to be alert closer to the heavily used Teton Park Road compared with the nearby quieter River Road. Furthermore, pronghorn foraged less at distances greater than 200 m from the Teton Park Road, while both species exhibited increased alert behavior when greater than 500 m from the Teton Park Road. Interestingly, 500 m is commonly cited in the literature as a threshold distance at which large predators (e.g., wolves and bears) avoid roads [36]–[38]. Pronghorn also demonstrated lower levels of movement at the Teton Park Road study site. These behavioral differences suggest that in contrast to the River Road, the area immediately adjacent to the Teton Park Road is of considerably lower perceived risk, despite the increased exposure to human activity and disturbance. In addition to effects of site, we also detected effects of group size on behavior, as pronghorn, and to a much lesser degree elk, appeared to feed more when in larger groups, suggesting potential fitness benefits from aggregating with conspecifics [39], [40]. Group size, however, was not a key explanatory variable in the analyses of alert or movement behavior for either species.

Our finding that elk did not exhibit the same pronounced increase in alert behavior, or indeed greater movement at the River Road, as demonstrated by pronghorn, is consistent with known differences in anti-predator behavior between the species. Elk are predominantly susceptible to predation from wolves and to a lesser extent coyotes and bears, and compared with the smaller-bodied, highly-responsive pronghorn, are less likely to respond or take flight when faced with perceived threat [41]. Previous studies have in fact demonstrated that elk exhibit highly variable behavioral responses to wolves that have been dependent on site, sex and physiological condition [33], [39], [40], [42]–[44], which could explain the comparatively variable effect of site in predicting alert behavior among groups of elk.

Reduced anti-predator behavior, as evident by diminished alert behavior and greater feeding along the Teton Park Road, concurs with Brown et al. [24] who found that ungulates did not consistently respond to human disturbance as a form of predation risk in GTNP. Earlier research has demonstrated that ungulates have the capacity to habituate to the presence of humans - even in urban environments - if exposed repeatedly to predictable, low-risk human activities [19], [22], [23], [25]. However, if reduced anti-predator behavior along Teton Park Road was driven solely by habituation or tolerance to human disturbance, we would expect to find similarly reduced responsiveness across sites with and without humans, particularly as the roads are in relatively close proximity to one another and the study animals can likely move freely between them. The greater responsiveness of ungulates along the River Road is therefore not entirely consistent with the idea of habituation along the Teton Park Road. One further explanation could be that ungulates do in fact perceive human activity as a predatory risk, but they cannot maintain continuous levels of alert behavior associated with the chronic disturbance experienced at the Teton Park Road [24], as would be predicted if animals were making risk allocation decisions [45], [46]. Yet if this were so, then we would have expected that periods with greater vehicle traffic at the quieter River Road would have resulted in greater responsiveness and reduced foraging. No such relationship, however, existed for either pronghorn or elk.

Our results also concur with recent studies that demonstrated disparities in the spatial distribution of predator and prey species as a function of human presence [26]–[28]. Whilst the Teton valley supports a variety of large predator species, during at least two years of the study (2007 & 2008), wolves denned within approximately 1 km of the River Road (Sarah Dewey, Wildlife Biologist GTNP pers. comm.), likely elevating their activity and increasing predation risk in the vicinity. Large carnivores are likely to avoid habitats in close proximity (<500 m) to comparatively busy roads such as the Teton Park Road that experience consistent levels of vehicle traffic throughout the daylight hours [38], [47]–[50]. However, it is important to note that we were unable to address the proportion of each species susceptible to predation along the two sections of road. Meanwhile, ungulate species can adjust their behavioral responses to human disturbance according to the degree of risk that they experience across both time and space [24], [25]. A recent study demonstrated that the highest level of vigilance exhibited by elk was associated with the hunting season on private and public lands, whilst the lowest was found in a neighboring national park during the summer when human presence was elevated, but the associated activities were relatively benign [18]. Human impacts on wildlife in natural and protected areas is therefore highly context-dependent and can vary dramatically depending upon the type of activity, predictability in patterns of use, and the behavioral sensitivity of species that are likely to be affected.

We conclude that the most parsimonious explanation of our results is that ungulates in GTNP can use predictable human presence along the Teton Park Road as a potential refuge from predation risk and adjust their behavior accordingly, further demonstrating the important conservation and management implications of understanding behavioral responses of wildlife to anthropogenic disturbance [51], [52]. These refuges are potentially strong attractors for prey populations and thus have the potential to drive trophic-mediated effects similar to those proposed by the reintroduction or extirpation of large carnivores [10], [11], [22]. Human activity could tip the “space-race” balance in favor of prey over predators and reduce the area of habitat that is available to large carnivores such as bears and wolves that are typically human-averse [17], [21], [27], [28]. Meanwhile herbivore populations could potentially increase population growth due to reduced predation, resulting in elevated impacts on sensitive habitats [22]. With an ever-growing demand for access to natural and protected areas, the indirect effects of human presence on wildlife will become more prevalent and it is essential that these impacts be better understood in order that effective conservation management can be balanced with the visitor experience.
